# SERT and BDNF polymorphisms interplay on neuroticism in borderline personality disorder

**DOI:** 10.1186/s13104-020-4924-6

**Published:** 2020-02-07

**Authors:** Valeria Salinas, Juana Villarroel, Hernán Silva, Luisa Herrera, Sonia Jerez, Alejandra Zazueta, Cristián Montes, Rodrigo Nieto, M. Leonor Bustamante

**Affiliations:** 1 Neurogenetics Clinic and Laboratory, University Neurology Center and Neurology Section, J.M. Ramos Mejía, Hospital, Faculty of Medicine, Universidad de Buenos Aires, Buenos Aires, Argentina; 2 Precision Medicine and Clinical Genomics Program, Translational Medicine Research Institute, Faculty of Biomedical Sciences, Universidad Austral-CONICET, Buenos Aires, Argentina; 3 University Psychiatric Clinic, Clinical Hospital, Universidad de Chile, Santiago, Chile; 4 Department of Psychiatry and Mental Health, North Division, Faculty of Medicine, Universidad de Chile, Av. La Paz 1003, Recoleta, Santiago, Chile; 5 Human Genetics Program, Biomedical Sciences Institute, Faculty of Medicine, Universidad de Chile, Santiago, Chile; 6 Program in Sciences and Engineering for Health, Universidad de Valparaíso, Valparaíso, Chile; 7 Department of Neuroscience, Faculty of Medicine, Universidad de Chile, Santiago, Chile

**Keywords:** Genetics, Personality, Five-factor model, Gene interaction, 5HTTLPR

## Abstract

**Objective:**

Genetic factors underlying different personality traits are not entirely understood, particularly how genes interact to modulate their effect. We studied 76 patients diagnosed with borderline personality disorder (BPD), characterized by extreme levels of personality traits, especially neuroticism (N), in which we genotyped two polymorphisms, the 5HTTLPR of the Serotonin transporter (*SERT*) gene, and the Val66Met of the Brain-derived neurotrophic factor (*BDNF*) gene.

**Results:**

We found an association with *SERT*, where S-allele carriers had significantly higher levels of N than L-homozygous. Furthermore, we found that the protective effect of L-homozygosity is only evident on A-allele carriers of the *BDNF* Val66Met polymorphism. Genetic constitution in *SERT* and *BDNF* seems to be important in neuroticism, the most relevant personality trait on BPD.

## Introduction

Inter-individual differences in personality can be at least partially accounted for by genetic factors. Personality traits are predictors of the development and course of mental disorders, and it is not clear whether this association is explained by shared genetic influences or by a different mechanism. It is estimated that the genetic underpinnings of personality traits are extremely complex. Nevertheless, understanding them is relevant to unravel the biological bases of vulnerability to personality disorders as well as other mental illnesses.

Borderline personality disorder (BPD) is characterized by affective dysregulation and impulsivity that lead to impairments in interpersonal, cognitive, behavioral and emotional functioning. It affects approximately 20% of consultants to psychiatric services, and its severity is determined mainly by impulsive behavior, including self-injury [1]. Several studies agree that the best framework to study BPD is the five-factor model of personality (FFM) [2], that describes personality in terms of Neuroticism (N), Extraversion (E), Openness to experience (O), Conscientiousness (C) and Agreeableness (A) [3]. In general, BPD can be understood as extreme levels of the FFM traits, specifically high levels of N, and low levels of A, C, and E [4]. The levels of personality traits, especially N, correlate with severity of impairment [5].

There is a moderate genetic contribution to normative personality traits. N is the personality trait that has the highest heritability [6]. On the other hand, the heritability of BPD has been estimated to be 40% [7]. Furthermore, there is a high degree of correlation between BPD and N, which is probably due to a shared genetic contribution [8]. Sharing of genetic risk factors has also been demonstrated for N and O and several psychiatric disorders including schizophrenia and mood disorders [9], which highlights the relevance of investigating genetic factors across conditions. For other personality traits, evidence suggests that the genetic contributions are smaller (with the exception of E), and that correlation with mental disorders is not explained by shared genetic factors.

In order to identify genetic factors explaining inter-individual differences in personality, we studied the association between a polymorphism in the serotonin transporter (*SERT*) gene known as 5HTTLPR (Serotonin transporter-linked promoter region) and one in the brain-derived neurotrophic factor (*BDNF*) gene (G196A, or Val66Met, rs6265), with FFM personality traits, in a sample of 76 individuals with borderline personality disorder (BPD). We focused on a defined group because we expect their characteristic personality profile would decrease the complexity of the phenotype and thus increase the power of our study. Furthermore, clinical variability among patients with BPD determines long-term outcome and therefore understanding its biological basis is also a relevant matter.

## Main text

### Methods and materials

#### Individuals and clinical measures

The study population sample was comprised of 76 patients diagnosed with BPD. All the cases were recruited at the University Psychiatric Clinic of Universidad de Chile. Structured interviews, SCID-I [10] and SCID-II [11], were used to identify DSM-IV disorders on Axis I and II. Exclusion criteria were history of mania or psychosis, present substance abuse, present depressive episode, and severe medical illness that could interfere with the clinical severity. All patients are of Chilean descent, and they all belonged to the same socioeconomic stratum (Stratum II) as defined by income and occupation [12].

A sample of 80 individuals with no mental disorders was recruited for comparing genotypic frequencies. They were screened using the MINI International Psychiatric Interview [13]. Any present or past mental disorder was an exclusion criterion, as well as any relevant medical disease as established by anamnesis. All controls are of Chilean descent, and they belonged to either II or III socioeconomic strata, meaning that they had either medium or low income and occupation levels.

Personality traits were studied according to the FFM, using the Spanish version of the NEO-FF Inventory [14]. The questionnaires were self-administered under the supervision of a research assistant (either a clinical psychologist or a psychiatry resident).

#### Laboratory procedures

DNA was extracted from peripheral blood leukocytes using commercially available kits and stored at − 80 ℃ until required. DNA integrity was evaluated using agarose gel electrophoresis, and quantification was carried out by microvolume spectrophotometry using a Nanodrop(R) equipment.

Genotyping of the rs6265 single nucleotide polymorphism (SNP) was carried out by RT-PCR, specifically using the TaqMan^®^ SNP Assays allele discrimination technique from Applied Biosystems. The components of the RT-PCR are in concentration and volume H_2_O 5.5 μl, Mastermix (2×) 2 μl, TaqMan Assay (20×) 0.50 μl, DNA (5 ng/Ul) 2 μl. Genotypes were obtained from the interpretation of the graphics using the StepOne software which is incorporated in the equipment.

5HTTLPR polymorphism genotyping was performed using the conventional PCR method. We used the primers 5′TCCTCCGCTTTGGCGCCTCTTCC3′ and 5′TGGGGGTTGCAGGGGAGATCCCG3′ [15]. The reaction was performed in total volume of 17 μl containing Taq polymerase and buffer; the equipment used is Thermal Cycler Operations, model PTC-100. PCR products were visualized by electrophoresis in 2% w/v agarose gels. PCR amplification allowed obtaining fragments or bands of 512 for L-allele and 469 bp for S-allele.

#### Statistical analysis

Univariate statistical analysis was performed using SPSS 13.0. Descriptive statistics included estimation of means and standard deviation (SD). Shapiro–Wilk test was used to establish normality of the data. Comparisons between groups defined by their genotype comparisons were made through ANOVA. Hardy–Weinberg equilibrium was studied by Goodness of Fit Chi square test. A *p*-value < 0.05 was considered statistically significant.

### Results

The patients’ sample was composed of 73.1% women, with a mean age of 34.2, with no differences in age distribution between females and males. The control sample was composed of 66.3% women, with a mean age of 33.7 years old.

Allelic frequencies for both polymorphisms showed no differences between patients and controls. For BDNF Val66Met polymorphism, the frequencies in patients were G = 0.79, A = 0.21; in controls, G = 0.76, A = 0.24. For 5HTTLPR, the frequencies in patients were L = 0.48, S = 0.52; and in controls L = 0.49, S = 0.51.

Mean scores (± SD) for personality traits for the whole group of patients were: N: 69.84 (± 14.73); E: 43.61 (± 15.79); O: 51.14 (± 14.85); C: 40.68 (± 11.33); A: 39.63 (± 12.07). For comparison purposes, mean values for the general population are 46–55 for each trait. Scores 56–65 are considered high, and > 65 very high, while scores 36–45 are considered low, and < 36, very low [16].

The scores for personality traits in the sample divided by the genotypes of the participants are presented in Table 1. For rs6265, the allelic and genotypic frequencies of the sample were in H–W equilibrium (*p* = 0.064). For 5HTTLPR genotypes, the allelic and genotypic frequencies of the sample deviated from H to W equilibrium (*p* = 0.055).

**Table 1:** Personality traits scores by BDNF Val66Met and 5HTTLPR genotypes in two groups

N = 76	Average score by BDNF Val66Met genotypes		*p* value	Average score by 5HTTLPR genotypes		*p* value
	G/G (N = 47)	A/A + A/G (N = 29)		L/L (N = 18)	S/S + L/S (N = 55)	
Neuroticism (M ± SD)	72.72 ± 14.56	66.16 ± 14.32	0.055	63.52 ± 17.39	72.35 ± 13.22	*0.022*
Extraversion (M ± SD)	43.6 ± 16.39	44.23 ± 14.67	0.864	45.21 ± 18.78	43.40 ± 14.67	0.665
Openness to experience (M ± SD)	53.25 ± 14.49	47.72 ± 15.04	0.155	50.22 ± 16.67	51.43 ± 14.38	0.765
Conscientiousness (M ± SD)	40.17 ± 11.77	41.51 ± 10.72	0.618	38.66 ± 11.15	41.31 ± 11.40	0.391
Agreeableness (M ± SD)	39.93 ± 12.11	39.13 ± 12.21	0.782	39.72 ± 11.04	39.60 ± 12.47	0.971

There were no significant differences observed among *BDNF* genotypes in any of the personality traits, although a trend was observed in N score (*p* = 0.055).

Significant differences (*p* = 0.022) were observed among *SERT* genotypes in the N score, but not among the other personality traits. The N score of S-allele carriers was on the “very high” range (score higher than 65), whereas L-allele homozygous had N scores on the “high” range (score between 56 and 65).

**Fig. 1:**
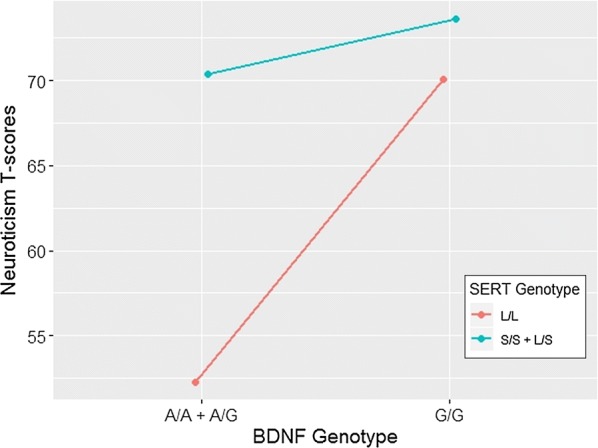
Neuroticism score of rs6265 A-allele carriers vs non-carriers, according to their SERT genotype

Figure 1 displays the interaction between the two genes, showing that L-allele homozygosity correlates with lower levels of N, only on A-allele carriers. S-carriers had higher levels of N, regardless of their *BDNF* genotype. In fact, S-carriers/GG individuals had 73.6 ± 13.8 (mean ± SD), while S-carriers/A carriers had 71.34 ± 11.79; on the other hand, G-allele homozygous had “very high” N scores regardless of their SERT genotype. GG/LL individuals had 70.1 ± 17.06, similar to GG/S-carriers (see above). These differences are not statistically significant, but this trend is interesting because the differences between subgroups are of potential clinical impact. In fact, the subgroup of LL/A-carriers has mean scores of N in the normal range (52.29 ± 11.84).

No interactions were observed with the other personality traits (data not shown).

### Discussion

In our sample of 76 patients with BPD, we observed higher levels of N in carriers of the S-allele of the *SERT* 5HTTLPR polymorphism. Furthermore, we observed a trend for an interaction between this polymorphism and rs6265 of *BDNF* gene, where the presence of the A allele of rs6265 have a trend to associates to lower levels of N only in L-allele homozygous.

Studying intra-class variability in psychiatric disorders is a useful strategy for understanding particularly complex phenotypes like personality traits. Of the five personality traits of the FFM, the one that has shown a more significant genetic contribution, and a more consistent relationship with BPD is N, and therefore our negative results with respect to other traits are not surprising. We have focused on N because of its impact on modulating the risk (of the general population) for suffering mental disorders and of BPD patients for having a worse outcome.

The S-allele of the 5HTTLPR has been widely considered as a risk factor for several psychiatric traits. However, studies of S-allele of the 5HTTLPR association with N have produced inconsistent results. One possible explanation for this is that its effect may be gender-specific [17]. Regarding BPD, several studies provide evidence of a modulating effect on the clinical features, where the S-allele determines worse outcome (for a systematic review see [18]).

As for *BDNF* rs6265, a meta-analysis [19] showed that A-allele carriers had significantly lower N scores than G-allele homozygous, in line with other studies suggesting that A-allele exerts a protective effect for psychiatric disorders [20]. However, this is not without controversy, because there is also evidence of association of the A-allele to impairment of neural structure and function, and higher levels of anxiety [21]. rs6265 has also been implicated in modulating the clinical features of BPD, including impulsive aggression [22], and susceptibility to environmental stress [23].

These candidate polymorphisms on *SERT* and *BDNF* genes have shown to modulate negative emotion processing [24, 25]. There are also biological and functional relationships between BDNF and serotonin, as BDNF promotes development of serotonergic synapses. Therefore, it is plausible that the effect of variants on these two genes interact to modulate personality traits, especially N which is closely related to anxiety. In fact, a study conducted on a large community-based sample found an interaction between rs6265 and 5HTTLPR where L-allele homozygous scored lower on N neuroticism only in the absence of the A-allele [21]. Interaction between rs6265 and 5HTTLPR has been observed in other traits related to anxious behavior, including conscientiousness (from the FFM), worry, cognitive reactivity and negative affect [26, 27].

The present study is unique in that we studied the effects of these two polymorphisms within the class of BPD and found an effect on the levels of N. Our results help to understand the differences between BPD patients that can have an impact on clinical outcome. Also, because there are shared genetic contributions to N and to BPD, this design can help understand N as a general trait.

## Limitations

We must acknowledge relevant limitations in our study. Our sample size is small, which leads to a loss of statistical power. However, we expect that our study is sufficiently robust because of the use of stringent exclusion criteria and a thorough clinical characterization. Interestingly, a recent study using factor analysis revealed two subclusters of N that have been named “Worry” and “Depressive Affect” and whose genetic influences can be separate [28]. To date, these two clusters have not been studied among BPD patients, or whether our candidate polymorphisms act differentially on these two features. This is certainly an aspect worth exploring in future studies.

Another limitation to acknowledge is that other variants of the promoter region of *SERT* gene were not studied. A SNP (rs25531, A/G) harbored in the variable region of the 5HTTLPR has been described that determines three main alleles: the S-allele, an L-A-allele, and an L-G-allele. The L-G has the same transcriptional efficiency as the S-allele, and therefore its presence may generate confounding results. While we did not include this analysis, we do not expect this to have an influence on our results, because the L-G allele has a very low frequency in Hispanic populations, and in fact a study found no L-G homozygous individuals [29].

Further studies must be performed including these and other genes in larger samples for a better understanding of the interplay of *SERT* and *BDNF* in Neuroticism, the most relevant personality trait in BPD.

## Data Availability

The data will not be shared in a public repository because during recruitment of subjects this authorization was not requested in the informed consent process. Individual requests for data access will be reviewed by the corresponding author.

## Abbreviations

5HTTLPR: Serotonin transporter-linked polymorphic region
A: Agreeableness
BPD: Borderline personality disorder
BDNF: Brain-derived neurotrophic factor
C: Conscientiousness
E: Extraversion
FFM: Five-factor model of personality
N: Neuroticism
SERT: Serotonin transporter
SNP: Single nucleotide polymorphism
